# Novel approach for accurate tissue-based protein colocalization and proximity microscopy

**DOI:** 10.1038/s41598-017-02735-8

**Published:** 2017-06-01

**Authors:** Mirjam I. Lutz, Carmen Schwaiger, Bernhard Hochreiter, Gabor G. Kovacs, Johannes A. Schmid

**Affiliations:** 1 Institute of Neurology, Medical University of Vienna, AKH 4J, Währinger Gürtel 18-20, 1090 Vienna, Austria; 2 Department for Applied Life Sciences, University of Applied Sciences, FH Campus Wien, Helmut-Qualtinger-Gasse 2, A-1030 Vienna, Austria; 3 Center for Physiology and Pharmacology, Department of Vascular Biology and Thrombosis Research, Medical University of Vienna, Schwarzspanierstraße 17, 1090 Vienna, Austria

## Abstract

Fluorescence colocalization microscopy is frequently used to assess potential links between distinct molecules; however, this method can lead to striking false-positive results and erroneous conclusions. Here we developed a novel approach with more sophisticated mathematical colocalization analyses together with visualization of physical proximity using fluorescence resonance energy transfer (FRET). To verify our results we used the proximity ligation assay (PLA). With these methods we could demonstrate that distinct neurodegeneration-related proteins either not or only rarely interact in human brain tissue.

## Introduction

Our interest focused on potential protein-protein interactions in various neurodegenerative diseases (NDDs). These are characterized by loss of neurons and the accumulation of proteins and are therefore termed proteinopathies of the brain^1^. The major NDD-proteins include tau, α-synuclein (α-Syn), and TDP-43. Each protein accumulates in a characteristic pattern, e.g. tau in primary tauopathies such as progressive supranuclear palsy (PSP), argyrophilic grain disease (AGD), corticobasal degeneration, and, together with Amyloid-β (Aβ), in Alzheimer disease (AD). α-Syn is characteristic for Parkinson disease (PD), dementia with Lewy bodies (DLB) and multiple system atrophy. And finally, TDP-43 in a neuropathological subtype of frontotemporal lobar degeneration (FTLD-TDP) and frequently as limbic TDP-43 proteinopathy associated to AD and other disorders^1^. In addition, co-existing proteinopathies, where accumulation of more than one type of neurodegeneration-related protein is seen in the same brain, have been frequently observed^1, 2^. Depending on the anatomical location (i.e., amygdala or substantia nigra) and disease, hyperphosphorylated-Tau (pTau) has been reported to colocalize to a varying degree with α-Syn and to be present in the same cells with TDP-43^3–6^. Furthermore, tau has been reported to colocalize with various other proteins in AD^7^, including Aβ, Aβ precursor protein (AβPP), complement factors, phospholipase C, various microtubule-associated proteins, ubiquitin and p62, as well as neurofilaments^8^. It has been suggested that α-Syn induces the formation of tau fibrils and depending on the α-Syn strain, tau and α-Syn synergistically promote the polymerization of each other^9, 10^. Indeed, observations in transgenic animal models^11^ and colocalization studies on human brain tissue^3, 5, 12^ implied an interaction between pTau and α-Syn. Furthermore, the synergistic relationship between AD-related pathology and TDP-43 could be suggestive of an interaction between related proteins^13^.

Most studies evaluating neurodegeneration-related proteins include minimal information on image acquisition, correlation coefficients or other objective measures, which are crucial for robust colocalization analysis, as we described recently^14^. Indeed, our previous studies revealed that a mere statistical analysis of pixel-intensity correlations can be misleading and sometimes even result in false positive colocalization estimates^14^. Furthermore, colocalization does not necessarily mean a physical interaction. To support the latter it is necessary to apply techniques that monitor the proximity of molecules, for instance by means of FRET microscopy, which has, to our best knowledge, not yet been used in human brain tissue-based research. In FRET, an energy transfer from an excited fluorophore (the “donor”) to a fluorophore with higher excitation and emission wavelength (the “acceptor”) is recorded. Since this quantum physical phenomenon occurs only at short distances between donor and acceptor of usually less than 10 nm, it is ideally suited to monitor physical proximity, which is in many cases indicative of an interaction^15–19^.

Based on our prior observations of partially false-positive colocalization results, this study investigated co-existing proteinopathies of the human brain in more detail using laser-scanning microscopy of brain sections stained for distinct protein aggregates. We compared frequently used image acquisition routines with some saturation of the detector with microscopy conditions that rule out any overexposure by adjusting the detector gain accordingly. In addition to advanced colocalization analysis, we applied 3-filter-based FRET microscopy^15, 17^ of the same view fields to visualize physical proximity of the stained proteins.

## Results

**Figure 1:**
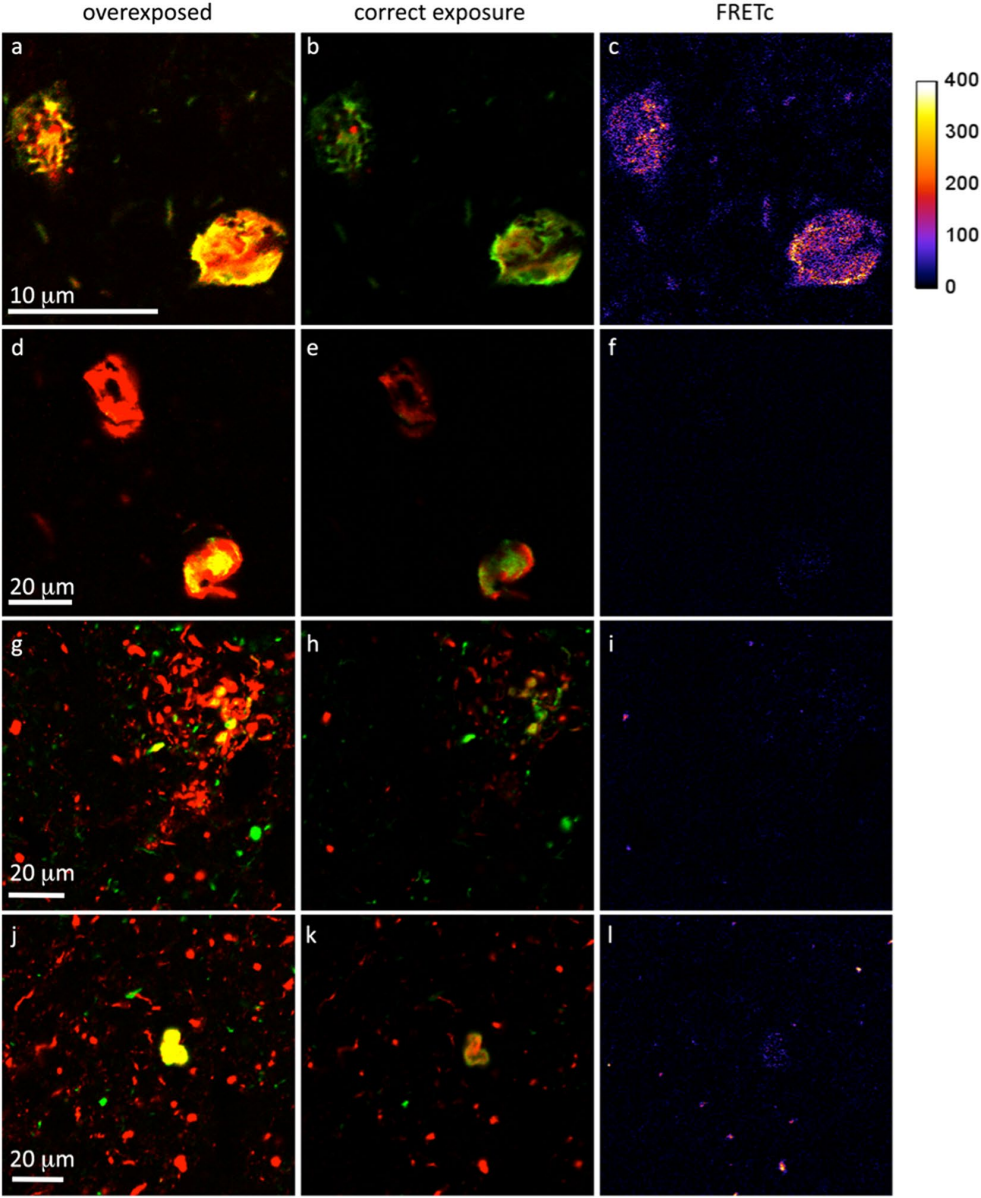
Confocal fluorescence images of double-immunolabeled human brain tissue sections. (**a**–**c**) double-labeling phosphorylated-tau (pTau) and pTau, positive control. (**d**–**f**) double-labeling ph-TDP-43 and pTau. (**g**–**l**) double-labeled α-syn and pTau. (**a**,**d**,**g**,**j**) over-exposed high illumination (apparent colocalization in yellow). (**b**,**e**,**h**,**k**) correct illumination without over-exposure. (**c**,**f**,**i**,**l**) corrected FRET (FRETc), x10 amplified in pseudo-color representation. A gradient color bar at the right indicates the numeric FRETc values for the different colors.

Microscopy under overexposure conditions often resulted in distinct yellow areas suggesting colocalization, while imaging of the same area without any saturation of the detectors revealed clearly distinguishable regions of green and red staining with minimal yellow color-mix (Fig. 1). Moreover, FRET microscopy revealed significantly positive areas only for control samples containing known protein-protein interactions, but not for the reported co-existing proteinopathies.

**Figure 2:**
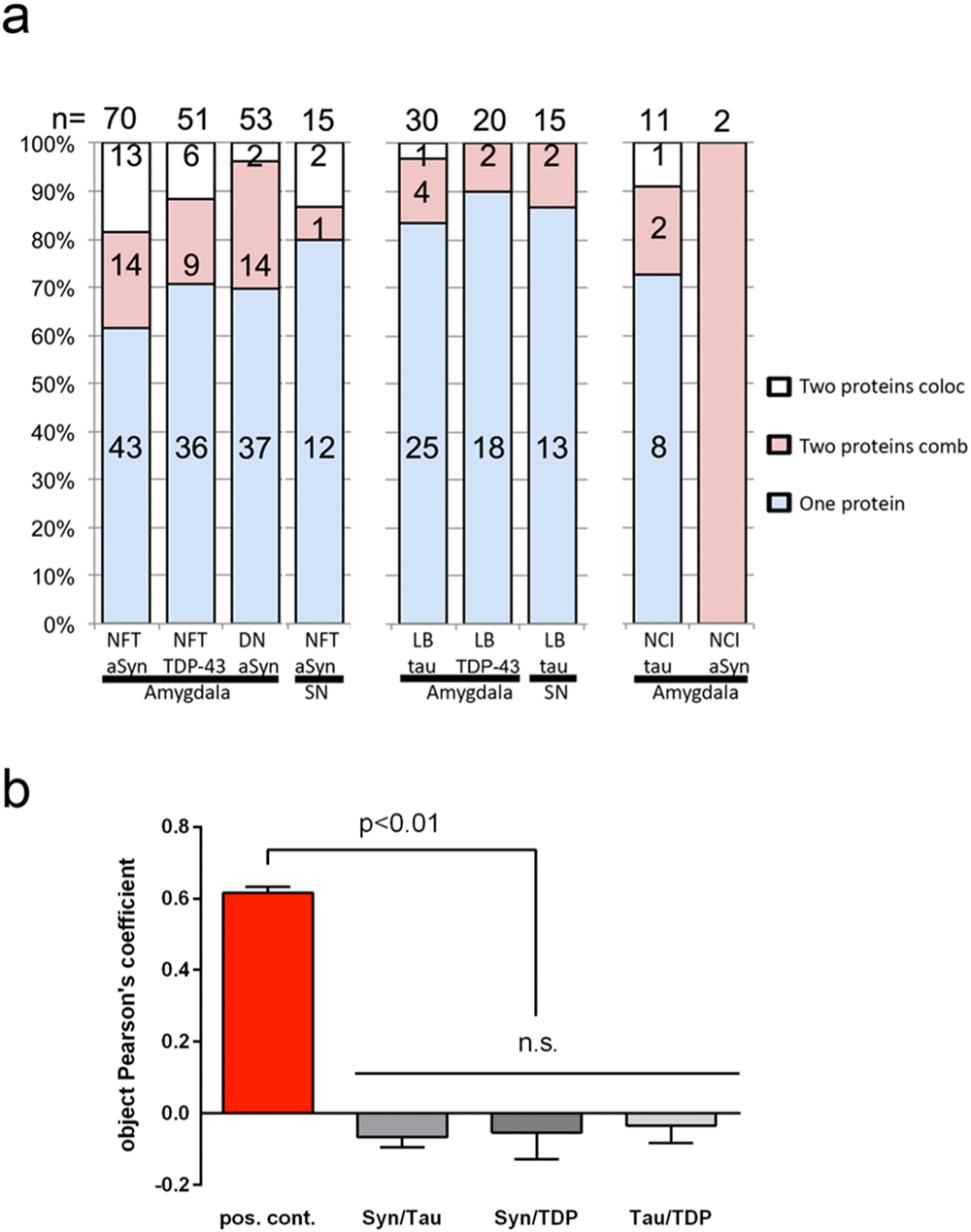
Comparison between conventional colocalization assessment and novel image analysis techniques. (**a**) Conventional assessment showing number (n) and percentage (%) of neuronal protein inclusions in different diseases in the amygdala and substantia nigra (SN). Lewy bodies (LB), neurofibrillary tangles (NFT), and TDP-43 positive neuronal cytoplasmic inclusions (NCI) were counted and the labels “one protein”, “two proteins combined” or “two proteins colocalizing” were given. Accumulations are grouped according to their primary protein and each column represents a combination with another protein. LB, α-Syn positive Lewy body; NCI, TDP-43 positive neuronal cytoplasmic inclusion; NFT, pTau positive neurofibrillary tangle; SN, substantia nigra. (**b**) Novel colocalization and FRET evaluation. Double stainings of α-synuclein/pTau (Syn/Tau), Syn/TDP and Tau/TDP have been analyzed with our new *ImageJ* macro and Pearson’s coefficient have been calculated for identified objects. A double Tau-staining served as positive control (Error bars represent SEM; Syn/Tau: n = 95; Syn/TDP: n = 16; TDP/Tau: n = 46; positive control (Tau/Tau): n = 185). Graphpad Prism™. ANOVA and Bonferroni post-testing revealed that the positive control differed significantly (p < 0.01) from the other stainings, but that the latter showed no significant difference between each other (n.s.).

Next, we attempted to improve colocalization analysis by establishing a high-content imaging routine combining FRET microscopy with object-based colocalization and pixel-intensity correlation. We created a novel macro for the *ImageJ* (*NIH*, https://imagej.nih.gov/ij/) software package (available on Github under: https://github.com/BHochreiter/ImageJ-FRET-and-coloc; see the Methods section for a more detailed description), which can be run in batch mode for a large number of images. It applies standard spectral bleed-through corrections to calculate corrected FRET images and uses threshold-based object recognition and segmentation for assessing colocalization^14^. For each identified object, Pearson’s correlation coefficient was calculated for the pixel intensities in the two channels, which outperforms whole-image-based intensity correlations. Additional parameters were computed, facilitating the evaluation of different degrees of colocalization in an automated and unbiased manner. These include the acceptor to donor ratio, a normalized, corrected FRET measure (NFRET), the colocalization area fraction and a color-mix coefficient. The latter reflects the extent of yellow intensity corresponding to mixing of red and green fluorescence and is defined as the ratio of donor to acceptor in the case of a higher intensity of acceptor, respectively the ratio of acceptor to donor in the case of a higher intensity of donor. Mean values between zero and one are obtained, with one representing a complete color-mix of the two channels (complete yellow corresponds to red and green colocalization), whereas zero represents the presence of only one marker (either green or red) in the respective region of interest. Using this automated image analysis approach, we analyzed 267 neuronal protein depositions such as pTau-positive neurofibrillary tangles (NFTs), α-Syn positive Lewy-bodies (LBs) and TDP-43 immunoreactive cytoplasmic inclusions. When we applied classical, standard image acquisition and analysis without eliminating saturated image regions (as shown in the left panel of Fig. 1), we detected 75 (28%) depositions containing two different NDD-proteins. Only 25 (9.3% of all) showed colocalization as assessed by the occurrence of a yellow color-mix and 50 (18.7% of all) neuropathological structures showed combined but not colocalizing proteins. This was dependent on the disease and the anatomical region examined (Fig. 2a). NFTs and phosphorylated (p)TDP-43 positive inclusions in the amygdala showed the most additional proteins (either α-Syn and TDP-43 or α-Syn and tau, respectively; sum of colocalizing and combined protein depositions 42, 34.7%, of 121 examined NFTs and in sum 5, 38.4%, of 13 examined TDP-43 inclusions) while the least were seen for α-Syn positive LBs (either tau or TDP-43, in sum 7, 14%, of 50 Lewy bodies in the amygdala and 2, 13.3%, of 15 in the substantia nigra, Fig. 2a). Evaluation of the images using our newly developed macro with object-based colocalization analysis provided more sophisticated quantitative values of Pearson’s intensity correlation per object, which would reach 1.0 in the case of perfect colocalization. For the co-staining of α-Syn and pTau we obtained an average object-Pearson coefficient close to zero. The same was observed for the combinations α-Syn/TDP and Tau/TDP, while the positive control of double-pTau staining showed a mean object-Pearson coefficient of 0.616 (Fig. 2b). An example of thresholding, segmentation of objects and calculation of corrected FRET signals is shown for a positive control sample (double pTau staining) in Supplementary Figure 1. Next, we set out to validate our approach with a well-defined cell culture model system. To that end, we transfected HEK293 cells with expression plasmids coding for spectrally distinct fluorescent proteins that localize specifically to mitochondrial or endoplasmic reticulum (ER) and tested different combinations with our FRET and colocalization analysis routine. This independent set of samples verified the suitability and robustness of our approach. Double staining of ER or mitochondria resulted in high object-Pearson coefficients close to 1, whereas a mitochondria/ER double-stain revealed a value of 0.04 (Supplementary Figure 2). Classical Pearson’s correlation analysis of whole images of these stains without object identification resulted in a misleadingly high Pearson coefficient in the range of 0.64 (data not shown and^14^).

Additionally, we verified our results using the proximity ligation assay (PLA), which monitors protein-protein interactions for ranges up to 40 nm^20^. Indeed, in this pilot study the PLA depicted rare positivity in tissues accumulating α-Syn and pTau, and pTau and pTDP-43, respectively (Supplementary Figure 3).

**Figure 3:**
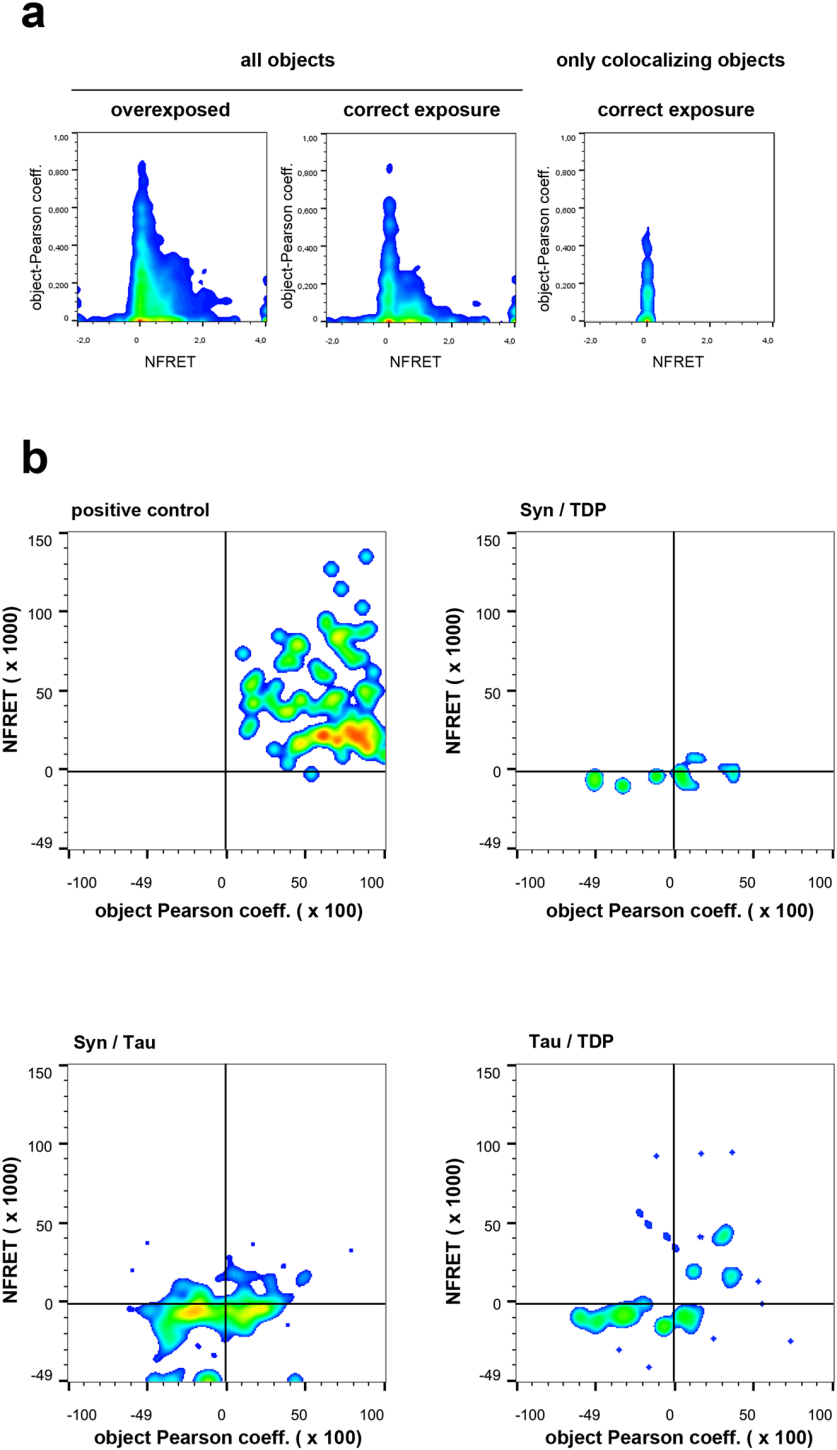
Combination of the novel colocalization analysis routine with FRET microscopy for the different neuropathologic stainings. (**a**) Visualization and analysis of FRET and colocalization with standard cytometry software (*FlowJo X*.*0*.*7*). Syn/Tau double stains were analyzed with our novel ImageJ macro and the results were further processed with MS-Excel 2013™, saved as comma-separated values (csv)-files and imported to the cytometry software. Smoothed pseudocolored density plots are shown for normalized, correct FRET values (NFRET) versus the object-Pearson coefficient. The impact of overexposure as compared to correct exposure is shown and the effect of restricting the analysis to colocalizing objects is depicted as indicated. (**b**) Cytometric evaluation of the different neuropathologic stainings. The positive control (pTau/pTau) exhibits an object population with clear positive values for NFRET and object Pearson coefficient (upper right quadrant). Double-stainings of distinct neurodegeneration-related proteins (Syn/TDP; Syn/Tau and Tau/TDP) show NFRET values close to zero and Pearson coefficients varying around zero.

A major advantage of our high-content imaging approach is the possibility to plot the analysis results of numerous objects and to derive more sophisticated statistics. This can be done in MS-Excel™ or similar spreadsheet programs, allowing for instance, the visualization of objects in 3-dimensional scattergrams, where the 3^rd^ parameter (e.g. the size of each detected object) is depicted as a diameter of the respective spot (bubble charts, Supplementary Figure 1). In addition, these rich datasets can be converted into file formats that are commonly used in cytometry applications so that intuitive visualization tools can be applied, including density or contour plots. By applying these cytometry analysis and visualization tools, we could demonstrate that infrequently occurring co-stainings of α-Syn and pTau in the brain sections that we investigated did not lead to any significant normalized FRET (NFRET) value, when proper image acquisition without overexposure of the detectors was done, followed by object identification and combined FRET/colocalization analysis (Fig. 3a). Evaluating the other co-stainings (Syn/TDP and Tau/TDP) similarly revealed an absence of any significant NFRET value and low object-Pearson coefficients, while a positive control (pTau/pTau) showed clearly positive values (Fig. 3b). A further advantage of cytometry-like analysis is that different logical gating algorithms and advanced statistics are possible, allowing detailed evaluation of the investigated parameters.

## Discussion

We can conclude that any robust assessment of colocalization or proximity of proteins or cellular structures requires more than simple microscopy of a few representative fields of view but rather the analysis of numerous regions of interest with strategies of high-content imaging and cytometry. We found that the combination of object-based colocalization analysis with pixel-intensity correlation according to Pearson^21, 22^ allows a reliable evaluation of colocalization. Nevertheless, it is evident that colocalization does not necessarily mean an interaction between two proteins. As a support for the latter, microscopy methods can be applied, which monitor a physical proximity of fluorophores, such as FRET microscopy. A positive FRET signal is an indication that the two fluorophores labeling the proteins of interest are within a range of less than 10 nm. However, it has to be stated that the absence of FRET does not rule out an interaction, as false-negative FRET results can occur if the distance of fluorophores is too far or the orientation inappropriate. On the other hand, a positive FRET signal is not inevitably proving a direct physical interaction of the two proteins, as a bridging molecule might lie in between. Still, a positive FRET value can be considered as strong indication for a functional interaction, no matter whether it is direct or indirect. In our study, we found that the parallel measurement of object-Pearson coefficients and normalized, corrected FRET values provides a solid evaluation of potential colocalization and physical proximity. Theoretically, the occurrence of FRET might influence Pearson coefficients by lowering donor fluorescence with a parallel increase of acceptor fluorescence. However, since FRET signals are usually rather low and FRET causes only small changes in donor or acceptor fluorescence, this effect is expected to be rather insignificant as compared to the extent of colocalization. Furthermore, colocalization analysis as assessed by the area fraction of colocalizing objects will be hardly influenced by FRET, as the minute alterations of donor and acceptor fluorescence are hardly affecting the process of thresholding and object identification.

For the specific question of co-existing proteinopathies in the human brain, we infer that there is no clear evidence for any significant colocalization and interaction or close proximity of neurodegeneration-related proteins on brain tissue slides, although rarely colocalization and physical vicinity can be seen. In spite of a considerable amount of “subjective” colocalization our unbiased approach revealed only a minor correct colocalization and a lack of unequivocal FRET signals. Importantly, we used antibodies detecting disease-associated protein forms including pTDP-43, pTau, and the 5G4 anti-αSyn antibody^23^, which might reveal different results as compared to antibodies detecting physiological protein-forms or other epitopes of disease-modified proteins. While simultaneous accumulation of different proteins in the brain is a common feature of these diseases^2^, it seems to occur more frequently in different cells or accumulate in distinct subcellular elements when present in the same neurons. Based on the application of the novel methods, including FRET and PLA in human brain tissue, our results suggest that cellular proximity of neurodegeneration-related proteins alone might not be sufficient to generate extensive concomitant proteinopathies. Ageing and neurodegeneration itself might initiate different pathogenic processes at the same time^2^, therefore, alternative or additional pathogenic pathways have to be considered to understand the phenomenon of mixed proteinopathies. Overloaded protein degradation systems might pose an adequate explanation. Involvement of protein degradation systems has been reported in AD^24^, prion diseases^25^, and synucleinopathies^26^. These studies suggest that distinct NDD-associated proteins use the same protein processing system. Thus, overloading of this system by one protein might directly influence and affect the life cycle of other proteins. Additionally, our recent community-based study indicated that neuropathologic alterations alone or together do not induce further pathologies with higher probability^2^. Based on these studies we propose there are at least three, potentially simultaneous mechanisms leading to concomitant proteinopathies: 1) protein-protein interactions or proximity; which, based on our present study and applied antibodies, seem to be rare and statistically non-significant; furthermore 2) overcharged protein degradation systems; and 3) unidentified factors generating synchronous and synergistic pathways for the development of proteinopathies within the same brain. Finally, the reported colocalizations of neuropathological proteins might simply be explained by inappropriate image acquisition with either insufficient resolution or saturated detector settings. Thus, the accurate application of colocalization studies is of utmost importance. Our new approach should be used as a tool for objective evaluation and interpretation of protein colocalization and/or interaction.

## Methods

### Brain samples

Human brain samples were collected at the Institute of Neurology, Medical University of Vienna. We obtained 13 female and 18 male human brains from the period 2007 to 2015. Patient ages ranged from 63 to 92 years (mean 81.62). This study was approved by the ethics committee of the Medical University of Vienna (number 2002/2016) and all experiments were performed in accordance with relevant Austrian guidelines and regulations, including the right to object from participating in scientific research. The manuscript does not contain information or images that could lead to an identification of the individual or which could violate any personal rights.

Demographic and clinical data are summarized in Table 1.

**Table 1:** Demographic data.

case	gender	age (in years)	neuropathologic diagnosis
**1**	f	90	LBD (B4) + LTDPP + AD (BB II)
**2**	f	88	LBD (B6) + LTDPP + AD (BB II)
**3**	m	88	LBD (B4) + LTDPP + AD (BB II)
**4**	m	72	LBD (B6) + LTDPP + AD (BB VI)
**5**	f	80	LBD (B4) + LTDPP + AD (BB VI)
**6**	m	66	LBD (B6) + LTDPP + AD (BB III)
**7**	m	80	LBD (B6) + LTDPP + AD (BB VI)
**8**	m	84	LBD (B4) + AGD + AD (BB III)
**9**	m	83	LBD (B5) + AGD + AD (BB II)
**10**	m	80	LBD (B5) + AGD + AD (BB III)
**11**	m	74	LBD (B4) + AGD + AD (BB II)
**12**	m	78	LBD (B6) + AGD + AD (BB IV)
**13**	f	83	LBD (B5) + AGD + AD (BB VI)
**14**	f	81	LBD (B5) + AD (BB III)
**15**	f	86	LBD (B4) + AD (BB IV)
**16**	m	77	LBD (B6) + AD (BB V)
**17**	m	82	LBD (B5) + AD (BB III)
**18**	m	70	LBD (B6) + AD (BB IV)
**19**	m	88	AD (BB III) + AMYGDSYNUC + AGD
**20**	m	63	AD (BB VI) + AMYGDSYNUC
**21**	m	84	AD (BB VI) + AMYGDSYNUC
**22**	f	85	AD (BB VI) + AMYGDSYNUC
**23**	f	93	AD (BB VI) + LTDPP
**24**	f	84	AD (BB V) + LTDPP
**25**	f	92	AD (BB IV) + LTDPP + AGD
**26**	m	87	AD (BB VI) + AMYGDSYNUC + LTDPP
**27**	f	88	AD (BB VI) + AMYGDSYNUC + LTDPP
**28**	f	79	PSP + LTDPP + LBD (B4)
**29**	f	80	PSP + LTDPP + LBD (B4)
**30**	m	82	PSP + LTDPP + AD (BB VI) + AGD
**31**	m	83	PSP + AGD + PSP + AD (BB V) + LBD (B5)

AD, Alzheimer disease-related neurofibrillary tangle pathology; AGD, argyrophilic grain disease; AMYGDSYNUC, α-synuclein inclusions restricted to the amygdala; B, Braak stages of α-Syn deposition; BB, Braak and Braak stages of NFT deposition; LBD, Lewy body disease indicating either PD or DLB; f, female; m, male; PD, Parkinson disease; PSP, progressive supranuclear palsy; LTDPP, limbic TDP-43 proteinopathy.

Brain tissue was fixed in formalin for approximately 21 days. After fixation in formalin, tissue blocks were embedded in paraffin. 5 µm thick sections were cut for immunohistochemical analysis.

### Case selection

All diseases were neuropathologically confirmed. The study included different mixed proteinopathies as listed in Table 1. Neurofibrillary pathology was Braak and Braak stage II or greater, Lewy pathology Braak 4 or greater. For the present study we used tissue blocks of amygdala and mesencephalon including the substantia nigra from each case.

### Double immunolabeling and Laser Confocal Microscopy

Paraffin embedded brain tissue was deparaffinized in xylene twice for ten minutes and rehydrated in graded ethanol until distilled water. Endogenous peroxidase was blocked in 0.9% H_2_O_2_/Methanol. Subsequently, epitopes were retrieved with the PT Link (Dako, Glostrup, DK) for 20 min at 95 °C (pH6, Envision flex target retrieval solution low pH, Dako, Glostrup, DK) followed by 1 min concentrated formic acid (Merck, 822254). To reduce the auto-fluorescence of aldehydes an additional step with two, two-minute incubations in 1% NaBH_4_ (Merck, 106371) in tris buffered saline (TBS) was performed. Unspecific binding of fluorochromes was prevented with 1 h blocking with 10% normal goat serum (NGS, Vector, S-1000) in TBS at room temperature (RT). Primary antibodies were used in various combinations diluted in 1% NGS for 1 h at RT. Antibody specifications are shown in Table 2. Slides were washed in TBS and incubated with fluorescent secondary antibodies Alexa Fluor488 and Cy3 for 1 h at RT and subsequently washed in TBS. Auto-fluorescence of lipophilic structures was blocked with 1% Sudan black B for 4 min at RT. Sections were mounted with Vectashield (Vector laboratories, Burlingame, CA). For negative control, the primary antibodies were omitted (data not shown). Slides were stored at −20 °C upon microscopic analysis.

**Table 2:** Antibody specifications. α-Syn, α-synuclein; dk, donkey; ms, mouse; rb, rabbit; pTau, phosphorylated tau; pTDP-43, phosphorylated TDP-43.

	Protein	Host	Clone	Phospho site	Company	Dilution
Primary	pTau	ms	AT8	p-S202/T205	Thermo Scientific	1:200
Primary	pTau	rb	—	p-S519/202	Assay Biotech	1:1’000
Primary	α-Syn	ms	5G4		Roboscreen	1:1’000
Primary	pTDP-43	rb	—	PS409/410	Cosmobio	1:2’000
Secondary	AlexaFluor 488	dk-anti- ms	A21121	—	Thermo Scientific	1:400
Secondary	Cy3	dk-anti-rb	111–165–144	—	Jackson ImmunoResearch	1:1’000

Combinations of primary antibodies were: 1) pTau (rabbit) and α-Syn (mouse), 2) pTau (mouse) and pTDP-43 (rabbit), 3) α-Syn (mouse) and pTDP-43 (rabbit), 4) pTau (mouse) and pTau (rabbit) (as positive control), and 5) α-Syn (mouse) and ubiquitin (rabbit) (as positive control). Additional information is provided in Table 2.

### Proximity ligation assay *in situ* (PLA)

Proximity ligation assay is an “*in situ*” detection method for protein-protein interactions. Fluorescence PLA was performed according to manufacturer protocol (Duolink *In Situ*, Red, Mouse/Rabbit, SigmaAldrich) with modifications for brain tissue samples as follows.

Brain tissue slides were deparaffinised in xylene for 10 minutes twice followed by rehydration in graded ethanol until distilled water. Subsequently epitopes were retrieved for 20 min at 95 °C in pH6 (Envision flex target retrieval solution low pH, Dako, Glostrup, DK) with the PT Link (Dako, Glostrup, DK) followed by 1 min concentrated formic acid (Merck, 822254). To reduce autofluorescence of aldehydes an additional step with 2 times 2 minutes 1% NaBH_4_ (Merck, 106371) in TBS was performed. Unspecific binding was prevented by 1 h blocking with 10% goat serum (Vector, S-1000) in TBS at RT. Primary antibodies as listed in Table 2 were incubated for 1 hour at RT. PLUS mouse and MINUS rabbit probe were mixed in 1:5 dilution in 1% normal goat serum 20 min prior to adding to the brain tissue. Slides were incubated with the probes in a wet chamber for 1 h at 37 °C. After thorough washing Ligation stock dilution in sterile water (1:5) with ligase was added and incubated for 30 min at 37 °C in a wet chamber and washing amplification stock was added to the tissue and incubated for 100 min at 37 °C in a wet chamber. After incubation slides were washed with buffer B. To block lipophilic autofluorescence we used 1% Sudan black B for 4 min at RT. Slides were mounted with Vectashield hard set (Vector laboratories, Burlingame, CA).

As negative controls brain tissues were treated without primary antibodies (data not shown). Slides were stored at −20 °C upon microscopic analysis.

### Image acquisition

All sections were assessed on a confocal Nikon A1 R+ System equipped with GaAsP detectors with 12-bit A/D conversion 12-bit detectors using a 60x plan apochromatic oil immersion objective (NA1.4). Alexa-488 labelled antibodies (FRET donors) were excited with a 488 nm Ar-laser with emission at 525 nm (band width 50 nm), Cy3-labels (FRET acceptors) were imaged with excitation at 562 nm and emission at 595 nm (band width 50 nm), and raw FRET signals were recorded with excitation at 488 nm and emission at 595 nm (band width 50 nm). Laser power was set to 2%.

### Analysis

Corrected FRET images were calculated according to the 3-Filter method^15, 17^. The spectral bleed-through of donor and acceptor fluorophores into the raw FRET channel was determined by imaging samples that were stained either with Alexa-488 or with Cy3 alone. A corrected FRET signal (*FRETc*) can then be calculated by subtracting the spectral bleed-through from the raw FRET signal according to the calculation:

$$FRE{T}_{C}(Youvan)=rawFRET-d\times DF-a\times AF$$

ref. 27 where:

*rawFRET:* raw FRET signal (acceptor emission at donor excitation)

*d*: donor correction factor (% of donor fluorescence observed in the raw FRET channel in a donor-only sample)

*DF*: donor fluorescence (donor emission at donor excitation)

*a*: acceptor correction factor (% of acceptor fluorescence observed in the raw FRET channel in an acceptor-only sample)

*AF*: acceptor fluorescence (acceptor emission at acceptor excitation)

This corrected FRET signal FRETc depends on the levels of fluorophores that are imaged at a specific site of the sample. For normalization between different sites or different samples, the FRETc value has to be normalized. We applied a commonly used normalization according to Xia *et al*.^28^ to calculate a normalized FRET value (NFRET) defined by the equation:

$$NFRET=\frac{FRE{T}_{C}}{\sqrt{DF\times AF}}$$

These calculations were included into a self-written macro for the *Fiji* version 1.51j (https://fiji.sc/) of *ImageJ* (*NIH*, https://imagej.nih.gov/ij/). The macro combines calculations of normalized, corrected FRET values (NFRET) with an object-recognition algorithm and computation of a Pearson-coefficient^22^ for each object. Object recognition was achieved by applying a threshold (using automated threshold algorithms, which are embedded into ImageJ) followed by standard “watershed” segmentation of adjacent, linked objects. The segmentation process is basically calculating a Euklidian distance map of thresholded objects, followed by the determination of ultimate eroded points and subsequent dilation of objects until they reach the neighbor object or the original dimension. In this way a one-pixel-wide separation is generated for objects that touch each other. The same threshold algorithm (e.g. the “moments” algorithm of ImageJ) was used for donor and acceptor channels, respectively. Objects with intensities above the threshold were converted to masks. A combination of the masks of the two channels was created using the “Max create” function of the ImageCalculator, revealing the combined regions of donor and acceptor. Using the “Min create” function of the ImageCalculator only overlapping (colocalizing) regions were obtained. The type of the thresholding algorithm has to be chosen carefully to match the respective staining or structure that has to be differentiated from background. While we also included the option of a manual, user-defined threshold into our macro code, we recommend using one of the automated threshold options for all the samples to prevent any bias from the user. Besides NFRET and the Pearson coefficient, the following parameters were quantified for each object: donor-, acceptor and raw FRET intensities; the bleed-through corrected FRET signal (according to Youvan^29^); the acceptor:donor ratio and a color-mix coefficient (the extent of yellow intensity in the case of mixing of red and green fluorescence in a region of interest). The later coefficient was defined as ratio of donor to acceptor in the case of a higher intensity of acceptor, or the ratio of acceptor to donor in the case of a higher intensity of donor, using an if/else loop in the macro. By that, mean values between zero and one are obtained, with one representing a complete color-mix of the two channels (complete yellow in the case of red and green), whereas zero represents the presence of only one marker (either green or red) in the respective region of interest. In addition, a colocalization area fraction was calculated by quantifying the colocalizing regions as percentage of the area of combined regions. The macro was executed in batch mode for a whole set of images for the same type of staining and the results were copied to MS-Excel 2013^TM^ for further analysis and data visualization. For evaluation of a higher number of objects we applied flow cytometry software (*FlowJo* X, www.flowjo.com). Numerical data (excluding text containing columns) were saved from MS-Excel 2013^TM^ as csv-files (comma-separated values), and imported into the *FlowJo* workspace to create smoothed pseudo-color plots. Converting the data to a flow cytometry format allows logical gating, definition of regions of interest as well as different forms of visualization and statistics for numerous objects.

### Code availability

The *Fiji/ImageJ* macro code that was newly developed and used for analysis (version 1.0) is freely available for use under ‘GNU General Public License version 3′ and can be found on Github under: https://github.com/BHochreiter/ImageJ-FRET-and-coloc. New versions will also be made available there in the future.

### Independent control experiments with fluorescent proteins

HEK 293 cells were transfected with expression plasmids coding for cyan- and yellow fluorescent proteins (CFP and YFP) localizing specifically to mitochondria or the endoplasmic reticulum and imaged as described in ref. 14. FRET and colocalization analysis was performed as described above.

### Statistics

Statistical evaluation was done with *GraphPad Prism™ 6*.*0* software. One-way analysis of variances (ANOVA) was applied with Bonferroni correction for multiple comparisons, as well as Kruskal-Wallis non-parametric tests (not assuming Gaussian distribution) with Dunn’s correction for multiple comparison. The p-value for statistical significance was set to 0.01.

## Electronic supplementary material

Supplementary information
