# Supplementary material for: Novel approach for accurate tissue-based protein colocalization and proximity microscopy
Source: Sci Rep. 2017 Jun 1;7:2668. doi: 10.1038/s41598-017-02735-8 (PMC5454019; doi:10.1038/s41598-017-02735-8)
Supplement: Supplementary file 1 — Supplementary information [file 41598_2017_2735_MOESM1_ESM.pdf]

Novel approach for accurate tissue-based protein colocalization and proximity microscopy

Mirjam I. Lutz*[1], Carmen Schwaiger*[1,2], Bernhard Hochreiter[3], Gabor G. Kovacs[#1], Johannes A. Schmid[#3]

[1] Institute of Neurology, Medical University of Vienna, AKH 4J, Währinger Gürtel 18-20, 1090 Vienna, Austria

[2] Department for Applied Life Sciences, University of Applied Sciences, FH Campus Wien, Helmut-Qualtinger-Gasse 2, A-1030, Vienna, Austria

[3] Center for Physiology and Pharmacology, Department of Vascular Biology and Thrombosis Research, Medical University of Vienna, Schwarzspanierstraße 17, 1090 Vienna, Austria

* equally contributing first author

# corresponding authors

Supplementary Figures

a

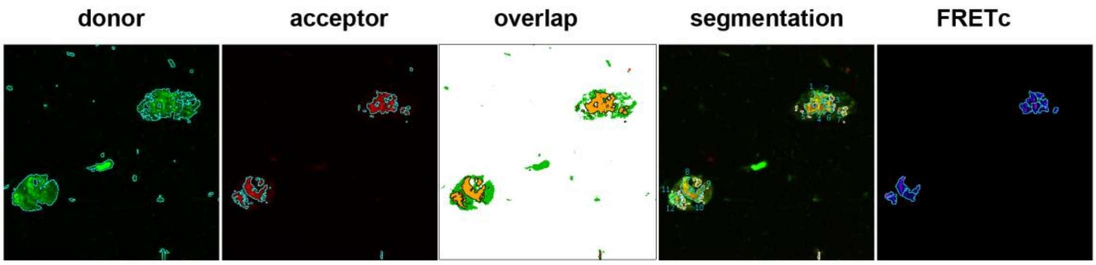

b

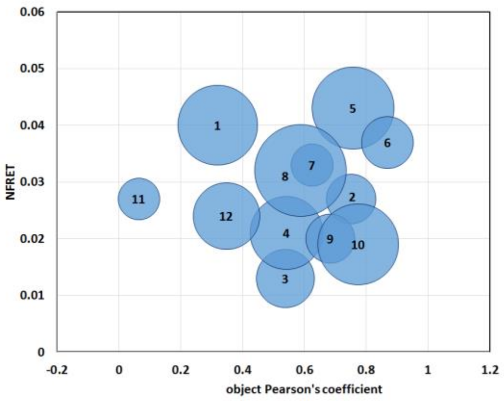

Suppl. Figure 1: Illustration of thresholding, segmentation and FRET calculation. a) Tau/Tau double-stains were imaged and analyzed as described in the Methods section. Borders of thresholded objects are depicted as lines in the donor and acceptor detection channel as indicated. The overlap image shows the colocalizing area in yellow with black boundary. The result of the watershed segmentation is shown in the next panel with numbers of the identified objects. A corrected FRET image (FRETc) is shown in pseudo-color mode. b) Results of NFRET and object Pearson coefficient calculation are depicted as bubble graph with the size of a recognized object represented by the area of the bubble and the numbers referring to the object numbers in a).

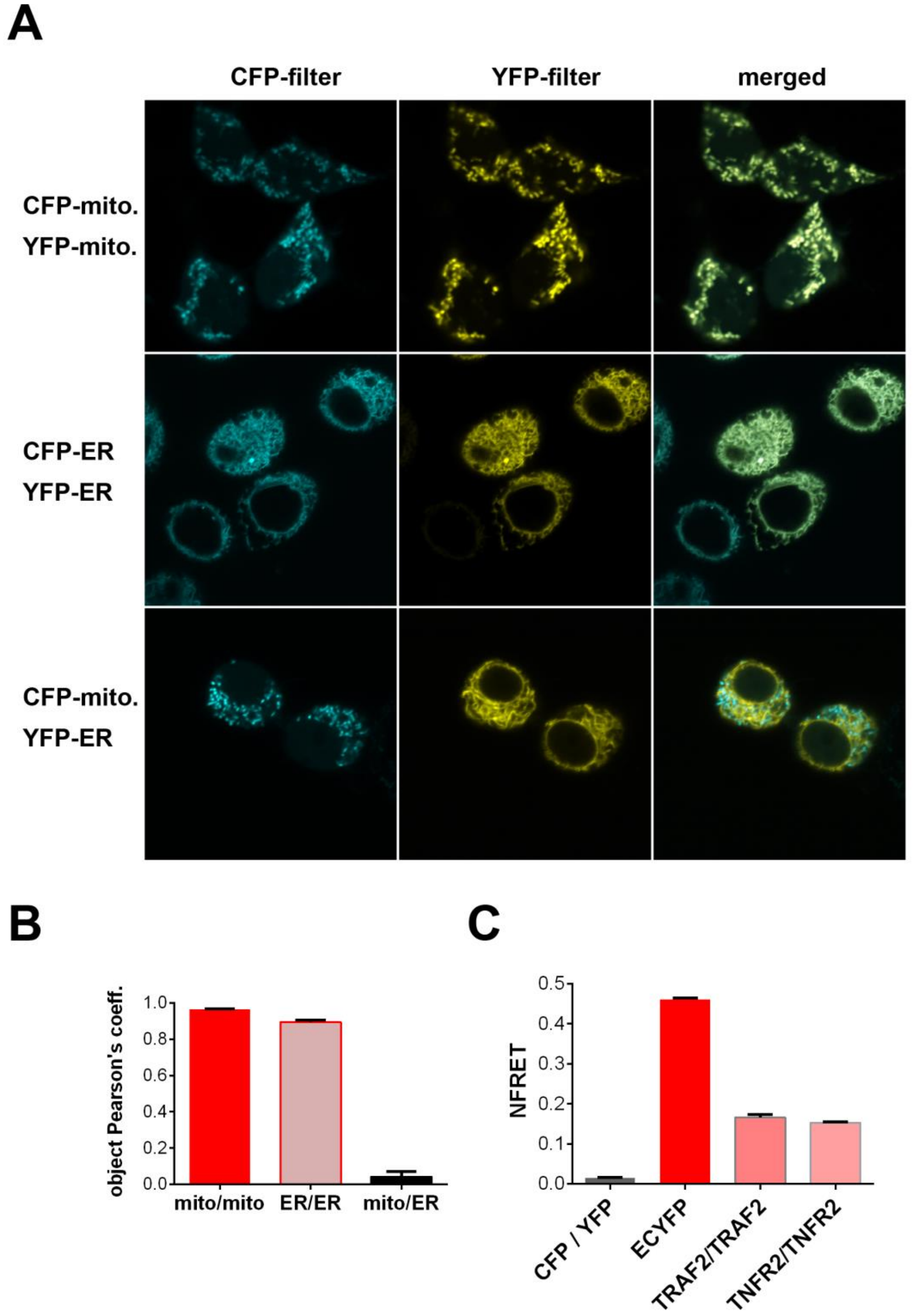

Suppl. Figure 2: Validation of the colocalization analysis in a well-defined cell culture model using spectrally distinct fluorescent proteins with known localization to mitochondria (mito) or endoplasmic reticulum (ER). A) HEK293 cells were transfected with expression constructs and imaged as described in Moser et al. 2017 (Ref 14). B) The Pearson's coefficient for identified objects is plotted (mean + SEM, n>162) for the compartments described in (A). C) Quantification of a normalized, corrected FRET values (NFRET) for HEK 293 cells transfected with a mix of non-interacting CFP and YFP proteins (CFP/YFP), a CFP-YFP fusion protein (ECYFP) with strong FRET and two different known homo-oligomer forming proteins: CFP- and YFP-TRAF2 (TRAF2/TRAF2) as well as CFP- and YFP-TNFR2 (TNFR2/TNFR2).

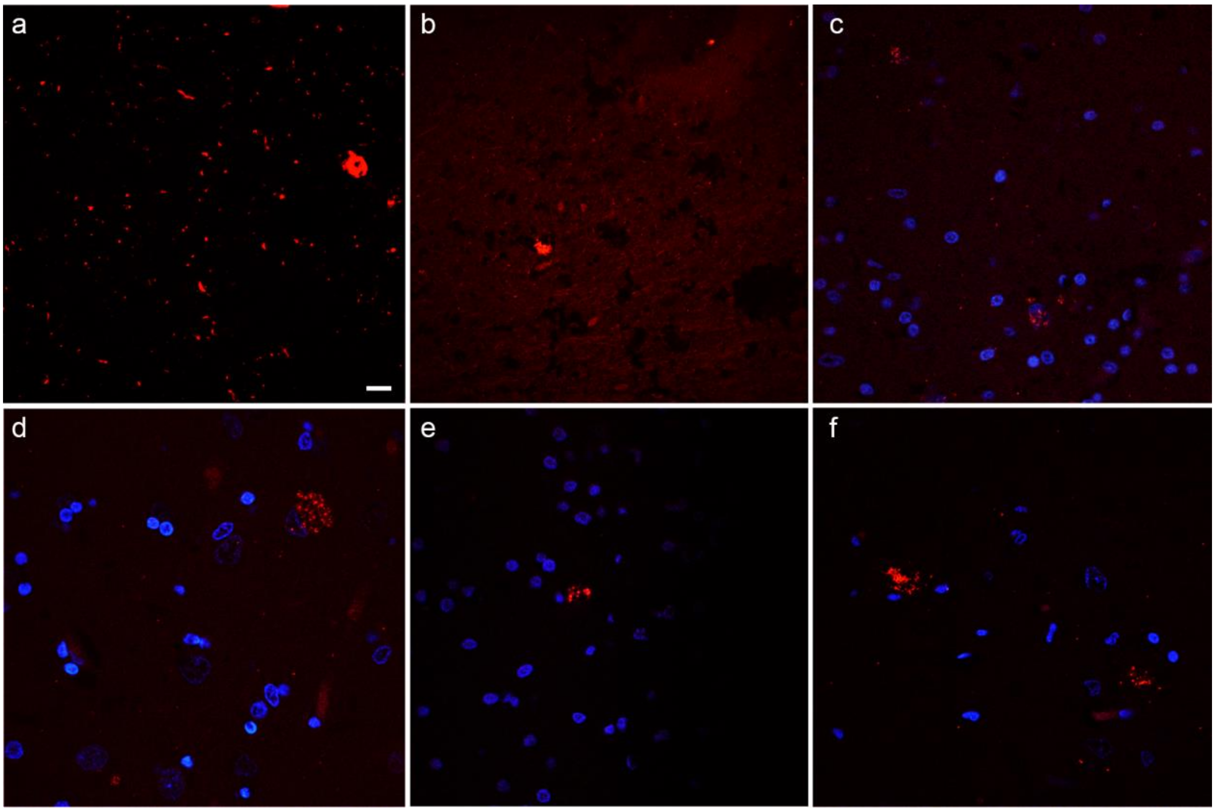

Suppl. Figure 3: Application of the novel proximity ligation assay. (a) double-labeling phosphorylated-tau (pTau) and pTau, positive control. (b) double-labeling α-syn and ubiquitin, positive control. (c) double-labeling ph-TDP-43 and pTau. (d-f) double-labeled α-syn and pTau. Bar = 10μm
